# Comparison of Sugars, Iridoid Glycosides and Amino Acids in Nectar and Phloem Sap of *Maurandya barclayana*, *Lophospermum erubescens*, and *Brassica napus*

**DOI:** 10.1371/journal.pone.0087689

**Published:** 2014-01-29

**Authors:** Gertrud Lohaus, Michael Schwerdtfeger

**Affiliations:** 1 Molecular Plant Science and Plant Biochemistry, Bergische University of Wuppertal, Wuppertal, Germany; 2 Albrecht-von-Haller-Institute, Systematic Botany, Georg-August-University of Göttingen, Göttingen, Germany; RIKEN PSC, Japan

## Abstract

**Background:**

Floral nectar contains sugars and amino acids to attract pollinators. In addition, nectar also contains different secondary compounds, but little is understood about their origin or function. Does nectar composition reflect phloem composition, or is nectar synthesized and/or modified in nectaries? Studies where both, the nectar as well as the phloem sap taken from the same plant species were analyzed in parallel are rare. Therefore, phloem sap and nectar from different plant species (*Maurandya barclayana*, *Lophospermum erubescens*, and *Brassica napus*) were compared.

**Methodology and Principal Findings:**

Nectar was collected with microcapillary tubes and phloem sap with the laser-aphid-stylet technique. The nectar of all three plant species contained high amounts of sugars with different percentages of glucose, fructose, and sucrose, whereas phloem sap sugars consisted almost exclusively of sucrose. One possible reason for this could be the activity of invertases in the nectaries. The total concentration of amino acids was much lower in nectars than in phloem sap, indicating selective retention of nitrogenous solutes during nectar formation. Nectar amino acid concentrations were negatively correlated with the nectar volumes per flower of the different plant species. Both members of the tribe Antirrhineae (Plantaginaceae) *M. barclayana* and *L. erubescens* synthesized the iridoid glycoside antirrhinoside. High amounts of antirrhinoside were found in the phloem sap and lower amounts in the nectar of both plant species.

**Conclusions/Significance:**

The parallel analyses of nectar and phloem sap have shown that all metabolites which were found in nectar were also detectable in phloem sap with the exception of hexoses. Otherwise, the composition of both aqueous solutions was not the same. The concentration of several metabolites was lower in nectar than in phloem sap indicating selective retention of some metabolites. Furthermore, the existence of antirrhinoside in nectar could be based on passive secretion from the phloem.

## Introduction

Floral nectars are aqueous, carbohydrate-rich solutions that are secreted by flowering plants to attract pollinators such as insects, birds and bats. Soluble metabolites which can be found in nectars mainly comprise mono- and disaccharides [1] and to a much lower extent amino acids [2], [3], [4]. Various nectars also contain multiple forms of other carbohydrates (i.e. melezitose) in minor concentrations [1].

Nectar sugar composition has often been related to the pollination syndrome of the plant species, where specific proportions of sucrose, fructose, and glucose may represent putative adaptations to dietary preferences of the respective pollinators. Flowers pollinated by hummingbirds, Old World bats, butterflies, moths and long-tongued bees tend to secrete sucrose-rich nectars, whereas those pollinated by perching birds, New World bats, short-tongued bees and flies tend to secrete hexose-rich nectars [5], [6], [7].

Nectar also usually contains an array of additional compounds, e.g. organic acids, lipids, proteins, antioxidants, inorganic ions, scents and other secondary compounds [3], [8]. The secondary compounds include alkaloids, phenolic substances, and iridoid glycosides which are commonly associated with herbivore defense [9], [10]. Although many plants produce nectar that is toxic or repellent to some floral visitors, the ecological significance of such toxic nectars is poorly understood [11]. Several hypotheses have been proposed to explain the functions of toxic nectar, including encouraging specialist pollinators, altering pollinator behavior, deterring nectar robbers, and/or preventing microbial degradation of nectar. For example, the floral nectar of *Catalpa speciosa* contains iridoid glycosides (e.g. catapol) that protect flowers from nectar robbers but not deter legitimate pollinators [9]. However, secondary compounds may also regulate the duration of pollinator visits and as a consequence the number of plants visited. Irwin and Adler [10] and Kessler et al. [12] demonstrated that the occurrence of the alkaloid gelsemine in nectar of *Gelsemium sempervirens* or nicotine in *Nicotiana attenuata*, respectively significantly decreased both frequency and length of pollinator visitations and reduced or increased pollen export [10], [12]. Most hypotheses about toxic nectars assume that the benefits of toxic nectar must outweigh possible costs. Alternatively, Adler [11] suggested that some toxic nectars may have no adaptive value, and that the presence of toxic compounds in nectar may be a result of their transport in the phloem and that these compounds passively ‘leak’ from the phloem into the nectar.

The site of nectar production, secretion, and release are the nectaries. These specialized organs occur in or around vegetative and reproductive organs. Nectaries can be extremely diverse with respect to their localization, their structure and probably even their secretion mechanisms [8]. Sieve tubes more or less directly supply secretory parenchyma cells called the ‘nectariferous tissue’ with pre-nectar [2], [13], [14] prior to nectar secretion. A detailed pathway has not yet been elucidated to describe how carbohydrates and other nectar components are uploaded from the phloem to the nectariferous tissue, metabolized and secreted to the outside. The carbohydrates can take several alternative routes. Some authors favor a symplasmic pathway, whereas others support transport through the apoplasm [13], [15]. The questions of where non-carbohydrate nectar compounds are produced, where and how they are added to the pre-nectar and how they are secreted still remain unanswered.

The species-specific differences in nectar composition could be explained in at least two physiological ways: (1) the metabolic pathways as well as the secretory process in nectaries control chemical composition and vary between species and/or (2) the composition of nectar reflects the chemical composition of the phloem, and varies between species [11]. Like nectar, phloem sap is an aqueous and sweet solution. The main sugar in the phloem sap is sucrose with concentrations around 1 M, whereas the amino acid concentration varies between 0.05 and 0.5 M [16], [17]. Some plant species also translocate oligosaccharides of the raffinose family or sugar alcohols [18], [19]. Many secondary compounds, including antirrhinoside (a common terpene-derived iridoid glycoside within the tribe Antirrhineae of the family Plantaginaceae), are also transported between plant tissues via the phloem in some plant species, e.g. *Asarina barclaiana* [19].

Differences in non-sugar nectar composition, including secondary compounds, may be caused by differences in phloem compounds that diffuse into nectar. Unfortunately, studies that measure both nectar and phloem composition of the same species are rare. One of the rare studies in this field is the comparison of the compounds in extrafloral nectars and in exsudates (mainly phloem exsudate) from cryopunctured fruits of cowpea plants [20]. In cowpea plants the sucrose:glucose:fructose weight ratio of nectar was about 1∶1∶1, whereas over 95% of phloem-exsudate sugar was sucrose. Also amino acid composition was different in nectar and phloem exsudate. Recently, Orona-Tamayo et al. [21] have shown that neither hexoses nor dominating nectar proteins were detected in the phloem exsudate of *Acacia cornigera* during nectar secretion from extrafloral nectaries, excluding the phloem as the direct source of these components.

In the study reported here, leaves, phloem sap and nectar of *Maurandya barclayana* (syn. *Asarina barclaiana*), *Lophospermum erubescens*, and for comparison also of *Brassica napus* were analyzed in parallel. *M. barclayana* and *L. erubescens* are members of the plant family Plantaginaceae and *B. napus* is a member of the Brassicaeae. These plant species were selected because it was known from former studies that *M. barclayana* and *B. napus* transport very different metabolites in the phloem [17], [18], [19]. The composition of nectar, the significance of phloem in supplying solutes to the nectar, and the possible metabolic transformations of soluble carbohydrates and nitrogen compounds were investigated. To evaluate which products of photosynthesis were withdrawn from the mesophyll cells of mature leaves for export by the phloem system leaf samples of the plant species were also analyzed. The origin and function of sugars, amino acids, and secondary compounds in nectar will be discussed.

## Materials and Methods

### Plant material

Seeds of *Maurandya barclayana (*syn. *Asarina barclaiana*) and *Lophospermum erubescens* were provided by the Botanical Garden of the University of Goettingen (Germany) and seeds of *Brassica napus* (genotype “Express”) were taken from the Institute of Plant Breeding, University of Goettingen (Germany). *M. barclayana* and *L. erubescens* are members of the plant family Plantaginaceae and *B. napus* is a member of the Brassicaeae. For each species six to ten plants were grown in a greenhouse. Each plant was potted in a single 2 L pot in compost soil and assayed when approximately three months old. Cultivation was carried out with a 15-h-light/9-h-dark cycle, a temperature regime of 23°C day/18°C night, and an irradiance of 250 to 350 µmol photons m^−2^ s^−1^. Mature leaves were used for the collection of phloem sap and leaf samples. Full opened flowers were taken for the collection of nectar.

### Collection of phloem sap with the laser-aphid-stylet-technique

**Figure 1:**
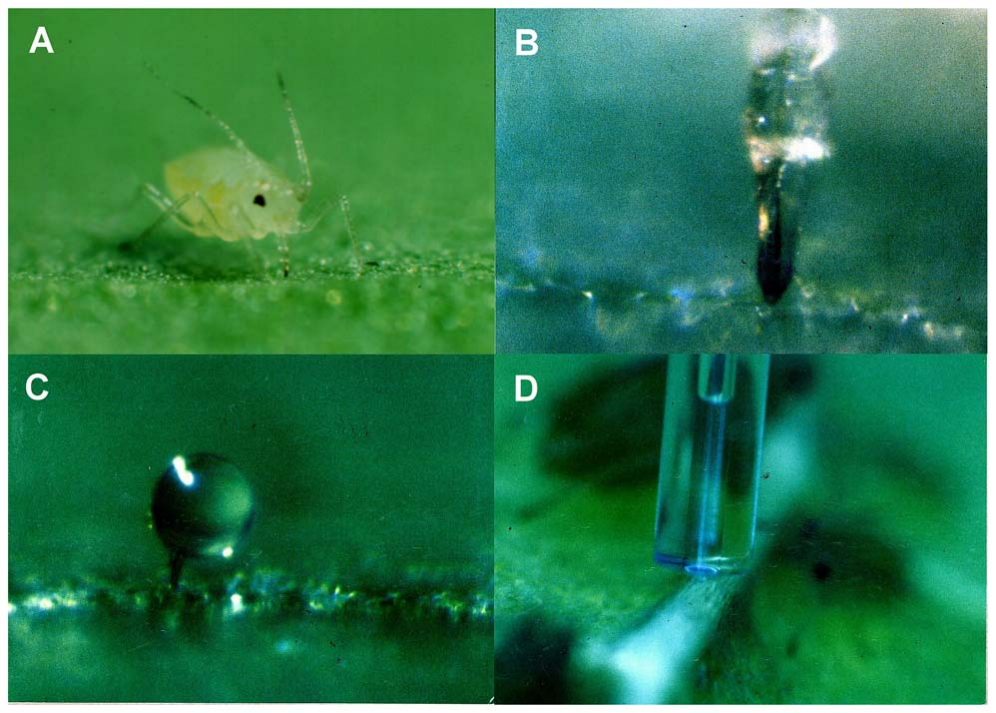
Collection of phloem sap with the laser-aphid-stylet-technique. (A) A feeding aphid of the family Aphididae. Aphids insert their very fine mouthparts (stylets) into phloem tissue, where they feed on phloem sap. (B) The stylet (the dark line in the middle of the labium) of the aphid in position for firing the laser. (C) Droplet of phloem sap exuding from a cut stylet. (D) Exuded sap collecting in a microcapillary

Phloem sap was collected with the laser-aphid-stylet technique [16], [22], [23]. Until now this is probably the best method to collect phloem sap from intact plants. Phloem sap was collected from mature leaves of flowering plants when approximately three months old. From each species 4 to 5 independent samples from different individuals were taken after 6 to 10 h of illumination. The laser-aphid-stylet-technique is generally very time consuming. Aphids of the family Aphididae were used for the experiments (Fig. 1A) and about 10 aphids were caged for about 5 h on the leaf. Their inserted stylets (Fig. 1B) were cut by a laser beam and the chance that phloem sap actually exuded from the severed aphid stylet was about 1 of 10 to 30. A successfully severed aphid stylet (Fig. 1C) exuded about 30 to 300 nL phloem sap. The exuded phloem sap was collected in a period of 1 to 4 h by placing a microcapillary (total volume 0.5 µL; Fig. 1D) over the cut end of the stylet using a micromanipulator. Because the equipment (laser, microscope) was blocked during this time only one sample could be collected at that time. The volume of the exudate was determined by measuring the length occupied by the solution. The leaves of the plant were illuminated during collection. Evaporation of the phloem sap was prevented by bringing the front edge of the capillary in close contact with the leaf surface and surrounding the end with a plastic cap. The humidity around the capillary was about 70%. Under these conditions no evaporation from reference capillaries was detectable. The samples were ejected into 50–100 µl of distilled sterile water and stored at −80°C.

### Collection of nectar

The nectar samples were taken from plants in parallel to phloem sap sampling. From each species 5 to 6 independent samples from different individuals were taken from fully-open flowers (about 24–72 h after anthesis) in the afternoon (after about 8 h of illumination). Nectar from *M. barclayana* (maximum 8 µl) and *L. erubescens* (maximum 30 µl) was extracted with micropipettes from single flowers. In the case of *B. napus* nectar was collected by means of a pair of binoculars in micro-capillaries (total volume 0.5 µL) and the nectar volume (less than 0.1 µL) was determined by measuring the length occupied by the solution [24], [25]. The nectar volumes correlated with the flower size. *M. barclayana* and *L. eruebscens* have tubular flowers and the corolla lengths were 44±3 mm (n = 9) and 70±5 mm (n = 9), whereas the flowers of *B. napus* were cross-shaped and much smaller (petal length 15 mm±2 mm; n = 9). Nectar samples were ejected into 1 mL of distilled sterile water. The humidity around the flowers was about 70%. Volume of nectar was recorded and samples were stored at −80°C.

### Water:chloroform:methanol extraction of leaves

The leaf samples were taken from plants in parallel to phloem sap sampling. From each species 3 independent samples from different individuals were taken after 8 h of illumination. After shock freezing in liquid nitrogen, 0.2 g leaf tissue was ground in a mortar and extracted on ice in 5 ml chloroform:methanol mixture (3∶7 v/v). The homogenate was then extracted twice with water. The aqueous phases were combined and evaporated in a rotatory evaporator. The dried residue was dissolved in 1 mL ultra-pure H_2_O (Millipore), syringe-filtrated (0.45 µm cellulose-acetate; Schleicher and Schuell, Germany) and stored at −80°C.

### Analysis of soluble carbohydrates

Sugars and glycosides in nectar, phloem sap, and leaves were assayed by HPLC according to Lohaus et al. [16]. An ion exchange column (CarboPAC10; Dionex Corp, Sunnyvale, CA, USA) was eluted isocratically with 60 mM NaOH (JT Baker Chemicals) (flow rate: 1 ml min^−1^) for 25 min. Sugars were detected by a pulse amperometric detector with a gold electrode (ESA, Model 5200, Coulochem II, Bedford, USA). Pulse setting was at 50, 700 and −800 mV for 400, 540 and 540 ms, respectively. Antirrhinoside was isolated from the plant species *Asarina barclaiana* and its chemical structure was determined by NMR [19]. Sugar standards (Sigma-Aldrich, Germany) and antirrhinoside standard were measured in parallel (0–500 µM) and for each carbohydrate a calibration curve was created. Sugar concentrations in nectar and phloem sap were calculated from the results of the analyses and the recorded volume of the respective sample. The evaluation of chromatograms was performed with the integration program Peaknet 5.1 (Dionex).

### Analysis of free amino acids

The assays were performed by HPLC (Pharmacia/LKB) according to Riens et al. [22]. After precolumn derivatization with o-phthaldialdehyde, the amino acid derivates were separated on a 4 mm particle size reversed-phase column (Merck, Darmstadt, Germany) with an acetonitrile gradient in 18 mM potassium phosphate, pH 7.1. The derivates were detected by fluorescence. Proline (a secondary amino acid) could not be detected with this method. Amino acid standards (Sigma-Aldrich, Germany) were measured in parallel (0–20 µM) and for each amino acid a calibration curve was created. The evaluation of chromatograms was performed with the integration program Peaknet 5.1 (Dionex).

### Statistics

Data for metabolite concentrations are shown as means (± SD). Statistical analyses were conducted using SPSS (v. 21, IBM Corporation). Significant differences (p≤0.05) were detected separately for each of the metabolite concentrations in the three species by one way ANOVA followed by multiple comparisons with TukeyHSD or Tamhane depending of the homogeneity or inhomogeneity of variances. Residuals of the models were analyzed by Kolmogorov-Smirnov and Levene's test to check for normal distribution and homogeneity of variances, respectively. If one of the assumptions of the ANOVA had to be rejected, Kruskal-Wallis rank sum test followed by Mann Whiney U test was conducted.

## Results

### Nectar concentrations

HPLC analysis of nectar of *M. barclayana* and *L. erubescens* showed that glucose, fructose, sucrose, mannitol, and antirrhinoside were present in both plants (Table 1). In both cases sucrose was the main soluble carbohydrate, but the concentration was about 1.5-fold higher in the nectar of *L. erubescens* than of *M. barclayana*. On the other hand the nectar of *M. barclayana* contained more glucose, fructose, mannitol and about 40 mM antirrhinoside. Very small amounts of raffinose were only found in single samples of nectar of *M. barclayana*. In contrast, nectar of *B. napus* contained nearly exclusively glucose and fructose, whereas the concentration of sucrose was in the low millimolar range (Table 1). In all plant species the total concentration of soluble carbohydrates in nectar was very high (between 894 and 1646 mM).

**Table 1:** Carbohydrate and amino acid concentrations in nectar, phloem sap and whole leaves from *M. barclayana, L. erubescens* and *B. napus*.

	Nectar [mM]		Phloem sap [mM]		Whole leaf^1^ [mM]		Whole leaf [µmol/g FW]
**a) ** ***M. barclayana***							
glucose	233±71.3	a	0.2±0.4	a	14.3±3.3	a	11.1±2.5
fructose	179±47.6	a	n.d.		11.8±3.4	a	9.0±2.6
sucrose	447±91.1	a	875±114	a	15.2±2.8	a/b	11.6±2.2
raffinose	0.8±0.7		26.9±3.9	a	1.2±0.2	a	0.9±0.2
stachyose	n.d.		34.3±9.6	a	1.6±0.2	a	1.2±0.2
mannitol	29.3±5.4	a	57.7±19.2	a	12.9±2.7	a	9.9±2.1
antirrhinoside	40.9±20.7	a	539±101	a	68.7±11.2	a	52.6±8.5
sum of carbohydrates	930±220	a	1533±107	a	126±17.8	a	96.2±13.6
sum of amino acids	0.8±0.2	a	106±12.9	a	14.3±0.7	a	10.9±0.5
sum of glucose equivalents in glu, fru, suc	1306	a	1750	a	56.5	a	
**b) ** ***L. erubescens***							
glucose	85.9±32.4	b	0.5±0.3	a	5.7±1.1	a	4.8±1.0
fructose	118±43.3	a	n.d.		6.6±1.7	a	5.6±1.4
sucrose	680±82.4	b	856±148	a	18.4±2.0	a	15.7±1.7
raffinose	n.d.		14.0±4.9	b	1.1±0.4	a	1.0±0.3
stachyose	n.d.		18.9±5.8	b	1.1±0.1	a	1.0±0.1
mannitol	6.0±1.3	b	43.5±19.0	a	14.8±3.9	a	12.6±3.3
antirrhinoside	4.3±1.4	b	32.5±13.5	b	16.2±2.1	b	13.8±1.8
sum of carbohydrates	894±154	a	965±145	b	64.0±9.5	a	54.6±8.1
sum of amino acids	0.2±0.1	b	81.1±23.4	a	7.9±0.2	b	6.8±0.2
sum of glucose equivalents in glu, fru, suc	1564	a	1712	a	49.1	a	
**c) ** ***B. napus***							
glucose	858±311	c	n.d.		6.6±1.1	a	5.7±0.9
fructose	782±323	b	n.d.		3.9±0.6	a	3.3±0.5
sucrose	5.5±2.7	c	947±110	a	5.7±0.5	b	4.9±0.4
raffinose	n.d.		n.d.		n.d.		n.d.
stachyose	n.d.		n.d.		n.d.		n.d.
mannitol	n.d.		n.d.		n.d.		n.d.
antirrhinoside	n.d.		n.d.		n.d.		n.d.
sum of carbohydrates	1646±437	b	947±145	b	16.1±1.7	b	13.9±1.4
sum of amino acids	2.0±0.4	c	315±81.7	b	22.9±3.1	a	19.7±2.6
sum of glucose equivalents in glu, fru, suc	1651	a	1894	a	21.9	a	
**d) all species**	**p (nectar)**		**p (phloem)**		**p (whole leaf)**		
glucose	0.001		0.272		0.061		
fructose	0.003				0.039		
sucrose	0.001		0.603		0.027		
raffinose			0.003		0.060		
stachyose			0.003		0.039		
mannitol	0.001		0.006		0.046		
antirrhinoside	0.001		0.002		0.024		
sum of carbohydrates	0.022		0.011		0.027		
sum of amino acids	0.001		0.008		0.027		
sum of glucose equivalents in glu, fru, suc	0.321		0.603		0.061		

1 Metabolite concentrations of whole leaves were estimated on the basis of leaf water contents and metabolite contents (µmol g^−1^ FW).

Abbreviations: FW  =  fresh weight; n.d.  =  not detectable (under detection limit of about 1 µM for each of the different carbohydrates and 0.1 µM for each of the different amino acids

Mean values from each five to six (nectar), four to five (phloem sap), and three biological (leaves) biological replications ± SD are shown.

Significant differences between plant species are indicated by different letters. The carbohydrate concentrations which were under the detection limit were not included in the post hoc test.

The amino acid concentrations were much lower than the carbohydrate concentrations and also differed between the plant species (Table 1). The nectar of *L. erubescens* contained the lowest concentration (0.2 mM) whereas the concentration in *B. napus* was tenfold higher (Table 1). Therefore, the ratio sum of carbohydrates to sum of amino acids was highest in *L. erubescens* and lowest in *B. napus* (Table 2). The percentage of each amino acid of the total amino acid concentration was also different between the three plant species (Table 3). The main amino acids in the nectar of *M. barclayana* were glutamate, glutamine, and glycine, in *L. erubescens* glycine, serine, and glutamate, whereas in *B. napus* glutamine was the main amino acid followed by glycine and asparagine.

**Table 2:** Different concentration ratios of carbon and nitrogen compounds in nectar, phloem sap and whole leaves from *M. barclayana, L. erubescens* and *B. napus.*

	Nectar	Phloem sap	Whole leaf
***a) M. barclayana***			
glucose/fructose	1.3	n.c.	1.2
sucrose/hexoses	1.1	4375	0.6
sum of carbohydrates/sum of amino acids	1162	14.4	8.8
sum of C/sum of N^1^	8528	142	90
***b) L. erubescens***			
glucose/fructose	0.7	n.c.	0.9
sucrose/hexoses	3.5	1712	1.5
sum of carbohydrates/sum of amino acids	4470	11.9	8.1
sum of C/sum of N	30646	109	74
***c) B. napus***			
glucose/fructose	1.1	n.c.	1.7
sucrose/hexoses	0.003	n.c.	0.5
sum of carbohydrates/sum of amino acids	823	3.0	0.7
sum of C/sum of N	3512	30	6.4

Abbreviations: n.c.  =  non-calculable because one sugar concentration was zero

1 Sum of C and sum of N were calculated by multiplication of the number of C or N atoms in the analyzed carbohydrates and amino acids with the sum of concentrations of carbohydrates and amino acids.

**Table 3:** Relative abundances of carbohydrates and amino acids in nectar, phloem sap and whole leaves from *M. barclayana, L. erubescens* and *B. napus*.

	*M. barclayana*			*L. erubescens*			*B. napus*		
	nectar	phloem	leaf	nectar	phloem	leaf	nectar	phloem	leaf
	[%]	[%]	[%]	[%]	[%]	[%]	[%]	[%]	[%]
**carbohydrates**									
glucose	25.0	0.0	11.4	9.6	0.1	8.9	52.3	0.0	40.7
fructose	19.2	0.0	9.4	13.2	0.0	10.3	47.4	0.0	24.0
sucrose	48.1	57.1	12.1	76.1	88.7	28.8	0.4	100	35.5
raffinose	0.1	1.8	1.0	0.0	1.4	1.8	0.0	0.0	0.0
stachyose	0.0	2.2	1.3	0.0	2.0	1.8	0.0	0.0	0.0
mannitol	3.2	3.8	10.3	0.7	4.5	23.2	0.0	0.0	0.0
antirrhinoside	4.4	35.2	54.6	0.5	3.4	25.2	0.0	0.0	0.0
**amino acids**									
glu	20.8	30.0	27.0	13.1	14.8	33.3	6.6	23.5	11.0
gln	19.4	28.9	33.1	6.8	9.2	21.9	26.0	19.1	47.7
asp	9.3	12.8	16.0	5.6	11.4	10.7	3.9	8.0	5.7
asn	0.7	0.5	0.4	5.3	1.4	0.7	9.4	4.5	5.2
ser	12.9	4.9	4.0	13.6	14.8	12.5	7.6	9.9	5.8
gly	14.8	3.3	0.5	29.8	9.3	2.2	9.6	1.2	7.9
ala	2.5	2.6	4.6	0.0	8.5	2.5	7.5	1.8	3.3
val	5.4	2.8	2.5	4.1	5.8	3.8	6.0	6.9	1.8
lys	1.6	0.4	0.2	10.2	2.3	0.3	5.2	6.4	1.4
arg and thr	5.0	6.8	1.2	3.1	5.4	2.9	7.4	4.9	7.1
gaba	0.0	0.0	1.3	0.0	1.5	3.6	0.0	0.1	0.1
other^a^	7.6	7.0	2.6	8.4	15.7	5.7	10.6	13.6	3.0

a Other amino acids means the sum of his, ile, leu, met, phe, trp, tyr which each percentages between 0.1% and 3%.

Abbreviations: gaba  =  gamma amino butyric acid

Abundances are given as the percentages of the total carbohydrate concentration and as the percentages of the total amino acid concentration, respectively.

### Phloem sap concentrations

The sucrose concentration in the phloem sap of all three species was very high (856–947 mM; Table 1). Sucrose was the only sugar in the phloem sap of *B. napus*, whereas both Plantaginaceae also contained smaller amounts of raffinose oligosaccharides (raffinose and stachyose), the sugar alcohol mannitol and the iridoid glycoside antirrhinoside. The antirrhinoside concentration in the phloem sap of *M. barclayana* was 539 mM and therefore antirrhinoside was the second major compound. Such high concentrations of antirrhinoside in the phloem sap of this plant species have already been reported (under the older synonyme “*Asarina barclaiana*”, [19]). No or only traces of glucose and fructose were found in the phloem sap.

The amino acid concentrations varied between 81 and 315 mM and the highest concentration was detected in *B. napus* (Table 1). The main amino acids in the phloem sap of all three species were glutamate, glutamine, aspartate, and serine (Table 3).

### Whole leaf contents

The metabolite contents of the whole leaves were measured in micromol per gram fresh weight (Table 1). Taking into account the water content of the leaves (*M. barclayana* 76.5%, *L. erubescens* 85.3% and *B. napus* 86.2%) metabolite concentrations of the whole leaves could be calculated as well (Table 1).

The carbohydrate concentrations in the whole leaves were much lower than in phloem sap and in nectar (Table 1). The leaves of all species contained glucose, fructose and sucrose. In *B. napus* no other soluble carbohydrates were found whereas *M. barclayana* and *L. erubescens* also contained raffinose, as well as stachyose, mannitol, and antirrhinoside. Antirrhinoside was the main soluble carbohydrate and made up nearly half of the total concentration of soluble carbohydrates of *M. barclayana* leaves. In leaves of *L. erubescens* antirrhinoside was the second major compound.

The amino acid concentration varied between 7.9 and 22.9 mM and the highest concentration was found in leaves of *B. napus* (Table 1). The main amino acids in the whole leaves of all three species were glutamate, glutamine, and aspartate with different percentages in the different plant species (Table 3). Glycine was also a dominated amino acid in leaves of *B. napus*.

### Comparison of the metabolite pattern between nectar and phloem sap

In each plant species, nectar contained at least glucose and fructose in addition to very different amounts of sucrose, whereas no hexoses were found in phloem sap (Table 1). In contrast, the total amount of soluble carbohydrates was in the same range in nectar and phloem sap (between 894 and 1646 mM), but in terms of glucose equivalents the amounts were always higher in phloem sap than in nectar (between 1.1 and 1.3; Table 4). The concentration of antirrhinoside was also 8–13-fold higher in phloem sap than in nectar (Table 4). The sugar alcohol mannitol was found in nectar if it was part of the compounds in phloem sap (Table 1) although the phloem concentration was always higher than the nectar concentration (Table 4). In contrast, in single nectar samples only small amounts of raffinose and no stachyose were found whereas both oligosaccharides were translocated in the phloem sap of *M. barclayana* and *L. erubescens* (Table 1). In all plant species the concentrations of amino acids were much lower in nectar (0.2–2 mM) than in phloem sap (81–315 mM) and the ratio of the sum of amino acids in phloem sap to nectar was very high (Table 4). Also the percentage of each amino acid of the total amino acid concentration was different in both fluids, e.g. the higher percentage of glycine in nectar is noticeable (Table 3). The ratio sum of carbon to sum of nitrogen increases from 30–142 in the phloem sap to 3512–30646 in nectar because of the lower amino acid concentrations in nectar in comparison to phloem sap (Table 3).

**Table 4:** Ratios of metabolite concentrations between phloem sap and nectar from *M. barclayana, L. erubescens* and *B. napus*.

ratio phloem sap/nectar	*M. barclayana*	*L. erubescens*	*B. napus*
sum of glu equivalents in glu, fru, suc	1.3	1.1	1.2
sum of amino acids	133	406	158
raffinose and stachyose	77	n.c.	n.d.
mannitol	2.0	7.3	n.d.
antirrhinoside	13.2	7.6	n.d.

Abbreviations: n.c.  =  non-calculable because one sugar concentration was zero; n.d.  =  non detectable; glu  =  glucose; fru  =  fructose, suc  =  sucrose.

### Comparison of the metabolite pattern between phloem sap and whole leaves

In a mature leaf most of the photosynthetic products are transferred from the source leaf cells into the phloem but the primarily assimilates and the transport forms are often different. In comparison to phloem sap the whole leaves contained hexoses as well as sucrose. It is well known that sucrose molecules, but no hexoses, are actively loaded into the phloem of different plant species including *M. barclayana* and *B. napus* [17], [18]. In *M. barclayana* and *L. erubescens* the percentage of antirrhinoside of the total concentration of soluble carbohydrates was higher in whole leaves than in phloem sap (Table 3). On the other hand, the concentration of raffinose and stachyose as well as the percentage of these oligosaccharides of the total concentration of soluble carbohydrates was higher in phloem sap than in whole leaves. Probably large parts of oligosaccharides in the phloem sap would be synthesized in the companion cells of the phloem and not the mesophyll cells [19], [26].

## Discussion

### Does nectar metabolite composition reflect that of the phloem sap?

The nectar of the three plant species *L. erubescens*, *M. barclayana* and *B. napus* contained very different concentrations of sucrose and hexoses (Table 1). The sucrose-to-hexoses concentration ratio differed from 3.5 in *L. erubescens*, 1.1 in *M. barclayana* to 0.003 in *B. napus* (Table 2). Comparatively little sucrose was detected in nectar of other members of the Brassicaceae [24]. The glucose-to-fructose ratios as well as the sucrose-to-hexoses ratios in the different nectars were independent from the phloem sugar composition, because in the phloem sap of all three species only sucrose was detected, whereas hexoses were almost completely lacking (Table 1 and 2).

The compositional differences between nectar and phloem compounds in these species imply that the phloem “pre-nectar” is modified to yield “mature” nectar (Table 1 and 2). Differences in nectar versus phloem composition may be due to metabolic processes in the nectaries, selective secretion of compounds into nectar and/or selective resorption from nectar into nectary tissue [2]. Carbohydrates are uploaded as sucrose from the phloem to the secretory tissue where they are stored as starch and/or further processed [8], [15]. During active secretion, sucrose is metabolized by invertases, which serve to produce hexose-rich nectars and create the required source–sink relationships [2], [27], [28]. An apoplastic invertase has recently been discovered in *Arabidopsis thaliana* that is required to create the sink status for active nectar secretion [28]. The hydrolysis of sucrose into glucose and fructose should yield a ratio close to one for each of the two hexoses. However, the ratio may deviate significantly from the expected 1∶1 in some species, e.g. *M. barclayana* (Table 2). This imbalance can be explained by prior cycling of sucrose through complex biochemical pathways before secreting into floral nectar [29] or by microbial degradation [30].

The oligosaccharide raffinose was only detectable in single samples of nectar from *M. barclayana* and was not detectable in the nectar of *L. erubescens*. In contrast, raffinose and stachyose were transported by the phloem sap of both plant species (Table 1). These differences indicate selective retention of oligosaccharides during nectar formation.

In all plant species the nectar was consistently much richer in sugars relative to amino acids than the phloem sap (Table 1 and 2), indicating also selective retention of nitrogenous solutes during nectar formation. However, higher amino acid concentrations in nectar correlated with higher amino acid concentrations in phloem sap (Table 1). Other evidence of selectivity in secretion is manifest in the differences in amino acid composition between phloem sap and nectar (Table 3). Similar results were shown for nectar from extrafloral nectaries and phloem exsudate from cryopunctured fruits from cowpea plants [20]. In this study it was shown that some ^14^C-labelled nitrogen compounds transferred directly to phloem and thence as such to nectar, while others have greatly impeded access.

### Nectar composition and pollinators

The composition of carbohydrates and free amino acids can affect the attractiveness of nectar for different pollinators. Concentration and composition of nectar sugars have often been correlated with specific responses of nectar visitors [5]. Based on the three carbohydrates predominating in most nectars, sucrose-to-(glucose and fructose) ratios have permitted designations of nectar type (sucrose-dominant and –rich, hexose-rich and –dominant) found to be favored by specific pollinators [5]. The nectar of *B. napus* is, like the most species of the Brassicaceae, dominated by hexoses and normally collected by short-tongued bees which prefer nectar rich in hexoses [5], [31]. *L. erubescens* is probably pollinated by hummingbirds [6] and had the most sucrose-rich nectar (Table 1 and 2). Nectar from flowers from other species in the tribe Antirrhineae visited by hummingbirds have shown similar percentages of sucrose, with an average of 76.2% [6]. *M. barclayana* is probably pollinated by bees like other species of the genera *Maurandya* [6]. The nectar of this species was sucrose-dominated but also contains high glucose and fructose concentrations (Table 1 and 2).

However, amino acids can also significantly affect the attractiveness of nectar [32], [33]. Birds and bats do not exclusively feed on nectar and can also gain nitrogen from other sources, whereas many adult insects feed only on nectar. Thus, amino acid concentration should be higher in insect- than in vertebrate-pollinated flowers [32]. This assumption corresponds with the result that the amino acid concentration was higher in the nectars of the insect pollinated plant species *M. barclayana* and *B. napus* than in *L. erubescens*, which is probably pollinated by hummingbirds [6]. On the other hand, the lower amino acid concentration could also be a result of the high nectar volume per flower in the case of *L. erubescens*. It could be assumed that in order to avoid strong nitrogen losses plant species with large nectar volumes reduce the concentration of nitrogen compounds.

### Origin and function of antirrhinoside in nectar

The floral nectar of both species of the tribe Antirrhineae, *M. barclayana* and *L. erubescens* contained the iridoid glycoside antirrhinoside. With regard to the question why the nectar of these plant species contains antirrhinoside, one possible explanation is based on adaptive processes. Floral nectar composition must fulfill at least two functions: a) attraction of specialist pollinators and b) protection from nectar robbers and microorganisms. It has been assumed that secondary metabolites fulfill the function to protect the nectar itself against nectar robbers [11], [34], [35]. Although nectar robbers do not necessarily reduce plant fitness, it is generally assumed that nectar consumption by nonmutualists may cause a loss of valuable resources. Some studies support the nectar robber hypothesis [9], [34] whereas other results indicate that even when nectar contains secondary compounds it may not serve as an effective barrier for nectar robbing (for review see [11]). Yet, it is not known if nectar robbing occurs in *M. barclayana* or *L. erubescens* like in other species of the tribe Antirrhineae. In a wild population of *Linaria vulgaris* nearly all open flowers were robbed [36] despite the fact that flowers of *L. vulgaris* contain high amounts of antirrhinoside [37]. In general, the function of iridoids in plants has largely been attributed to defense against herbivory by insects [38] and the concentration of antirrhinoside can reach up to 20% of the dry weight of buds, flowers or young leaves of *Antirrhinum majus* [39]. If one of the functions of antirrhinoside is to serve as a defensive compound, then it is not unexpected that there are higher contents in developing sink tissues. However, the function of antirrhinoside is still unknown [39].

Secondary compounds in nectar might be synthesized in the nectaries themselves. This would make an adaptive function of these compounds in nectar to pollinators or nectar robbers highly likely. But secondary compounds can also be derived directly from the phloem, thus suggesting a function that is not necessarily related to their appearance in the nectar [11], [35]. On the other hand the composition of compounds in phloem sap is influenced by the composition in leaves. Leaves of *M. barclayana* and *L. erubescens* contained high amounts of antirrhinoside, which was transported in phloem sap and was also detectable in nectar (Table 1). Therefore, a possible explanation for the presence of antirrhinoside in nectar could be that nectar may simply reflect phloem composition. In both plant species a downhill concentration gradient for antirrhinoside between phloem sap and nectar exists (Table 4) and passive secretion is feasible. In contrast, current studies about nectar proteins (nectarins) have reported different results [21]. In *Acacia cornigera* neither invertase nor other dominating nectar proteins were detected in the petiole exsudates (phloem exsudates), excluding the phloem as the direct source of major nectar components [21]. Also an extracellular invertase in *Arabidopsis* flowers is exclusively expressed in nectaries [28]. Probably in the case of nectarins or other regulatory compounds in nectar there is a stronger metabolic contribution of the nectary itself than in the case of carbohydrates and amino acids.

Future research should include the analyses of other secondary metabolites or proteins of both nectar and phloem sap to identify nectar compounds that originate exclusively from the nectar itself.
